# Cytotoxic compounds from the leaves and stems of the endemic Thai plant *Mitrephora sirikitiae*

**DOI:** 10.1080/13880209.2020.1765813

**Published:** 2020-06-01

**Authors:** Natthinee Anantachoke, Duangporn Lovacharaporn, Vichai Reutrakul, Sylvie Michel, Thomas Gaslonde, Pawinee Piyachaturawat, Kanoknetr Suksen, Samran Prabpai, Narong Nuntasaen

**Affiliations:** a Department of Pharmacognosy, Faculty of Pharmacy, Mahidol University, Bangkok, Thailand; b Department of Chemistry and Center of Excellence for Innovation in Chemistry, Faculty of Science, Mahidol University, Bangkok, Thailand; c Produits Naturels, Analyse et Synthèse, UMR CNRS 8038 CITCOM, Faculté de Pharmacie de Paris, Université Paris Descartes-Université de Paris, Paris, France; d Department of Physiology, Faculty of Science, Mahidol University, Bangkok, Thailand; e CP FOODLAB Co., Ltd, Science Park, Pathum Thani, Thailand; f Department of National Parks, Wildlife and Plant Conservation, Ministry of Natural Resources and Environment, The Forest Herbarium, Bangkok, Thailand

**Keywords:** Annonaceae, plant metabolite, alkaloid, lignan, diterpenoid, cytotoxicity, anticancer agent

## Abstract

**Context:**

*Mitrephora sirikitiae* Weeras., Chalermglin & R.M.K. Saunders (Annonaceae) is a plant endemic to Thailand. Its constituents and their biological activities are unknown.

**Objective:**

Isolation and identification of the compounds in the leaves and stems of *M. sirikitiae* and determination of their cytotoxicity.

**Materials and methods:**

Methanol extracts of the leaves and stems of *M. sirikitiae* were separated by chromatography, and spectroscopic methods were used to determine the structures of the components. The cytotoxicity of the extracts and pure compounds was evaluated using the sulforhodamine B assay with several cell lines. The cells were treated with the compounds at concentrations of 0.16–20 µg/mL for 48 or 72 h.

**Results:**

The investigation of the extracts of *M. sirikitiae* leaves and stems resulted in the isolation of a new lignan, mitrephoran, and 15 known compounds. Among these compounds, 2-(3,4-dimethoxyphenyl)-6-(3,5-dimethoxyphenyl)-3,7-dioxabicyclo[3.3.0]octane, ciliaric acid, 6-methoxymarcanine A, and stepharanine were isolated from this genus for the first time. The alkaloids liriodenine and oxoputerine exhibited strong cytotoxicity against all tested cells (IC_50_ values of 6.59–11.02 µM). In contrast, magnone A, 3′,4-*O*-dimethylcedrusin, and 6-methoxymarcanine A inhibited the growth of some of the tested cells (IC_50_ values of 2.03–19.73 µM). Magnone A and 6-methoxymarcanine A showed low toxicity for Hek 293 cells (IC_50_ >20 µM).

**Discussion and conclusions:**

*M. sirikitiae* is a source of cytotoxic lignans and alkaloids. Among the cytotoxic compounds, magnone A and 6-methoxymarcanine A are potentially useful lead compounds for the further development of anticancer agents because of their selective inhibitory effects on cancer cell lines.

## Introduction

Cancer is one of the major causes of morbidity and mortality worldwide. The International Agency for Research on Cancer (IARC) has estimated the incidence of cancer, and its mortality rate to be 18.1 million new cases and 9.6 million deaths in 2018 alone. One-third of global cancer cases are lung, breast, and colorectal cancers, and these are the top five causes of cancer-related deaths (Bray et al. 2018). Chemotherapy is the backbone of treatment for many cancers at different stages, complementing surgery and radiotherapy. However, cancer cells frequently develop resistance to chemotherapeutic drugs, limiting the effectiveness of treatment. Thus, the identification of new effective anticancer compounds is needed. A number of anticancer drugs are derived from natural sources including plants, microorganisms, and marine organisms (Bailon-Moscoso et al. 2017). Plants are important sources of biologically active compounds and promising chemotherapeutic agents. As part of our on-going study of bioactive compounds obtained from plants, we have investigated the cytotoxic compounds present in a variety of plants in Thailand. Interestingly, our *in vitro* screening of the cytotoxic activity of plant extracts indicates that those from *Mitrephora sirikitiae* Weeras., Chalermglin & R.M.K. Saunders (Annonaceae), known as Mahaphrom Rachini in Thai, have substantial cytotoxic activity. *M. sirikitiae* is a plant endemic to northern Thailand. It was first discovered on a mountain peak at an altitude of 1100 m in the Mae Surin Waterfall National Park, Mae Hong Son Province in 2004 (Weerasooriya et al. 2004). However, to date, phytochemical and biological studies of this plant have not been reported.

Plants in the genus *Mitrephora* have been reported to be a rich source of secondary metabolites including alkaloids from *M. teysmannii* Scheff., *M. diversifolia* Miq., *M. glabra* Scheff., *M. thorelii* Pierre, and *M. vulpina* C.E.C.Fisch. (Lee et al. 1999; Yu et al. 2005; Deepralard et al. 2007; Ge et al. 2008; Li et al. 2009; Mueller et al. 2009; Moharam et al. 2010); diterpenoids from *M. celebica* Scheff., *M. tomentosa* Hook. f. & Thomson, *M. glabra*, *M. thorelii*, *M. teysmannii*, and *M. alba* Ridl. (Zgoda-Pols et al. 2002; Supudompol et al. 2004; Li et al. 2005, 2009; Deepralard et al. 2007; Meng et al. 2007; Rayanil et al. 2013); lignans from *M. teysmannii*, *M. vulpina*, and *M. alba* (Deepralard et al. 2007; Moharam et al. 2010, 2014; Rayanil et al. 2013); dihydrobenzofuran lignans from *M. teysmannii* and *M. wangii* generic HU (Rayanil et al. 2016; Sanyacharernkul et al. 2016; Jaidee et al. 2018); lignanamides from *M. thorelii* (Ge et al. 2008), phenolic compounds and flavonoids from *M. teysmannii* (Deepralard et al. 2007); and polyacetylenic acids/esters from *M. celebica*, *M. glabra*, and *M. teysmannii* (Zgoda-Pols et al. 2001; Li et al. 2009; Rayanil et al. 2016). Natural compounds discovered from plants in this genus exhibit many biological effects, including antimicrobial (Zgoda-Pols et al. 2001, 2002; Li et al. 2005, 2009), antifungal (Sanyacharernkul et al. 2016), antimalarial (Mueller et al. 2009), antitumor (Meng et al. 2007), cytotoxic (Li et al. 2005, 2009; Rayanil et al. 2013), antiplatelet activating factor (Moharam et al. 2010), and *α*-glucosidase inhibitory activities (Rayanil et al. 2016).

In the present study, our cytotoxic activity screening of the crude methanol extracts and fractions of *M. sirikitiae* leaves and stems demonstrated that this plant exhibits moderate to potent cytotoxicity against many cancer cell lines. Therefore, we report herein the lignans, dihydrobenzofuran lignan, alkaloids, and diterpenoids isolated from the methanol extracts of leaves and stems of *M. sirikitiae*, together with their cytotoxic effects on the cancer cell lines P-388 (mouse lymphoid neoplasma), KB (human oral nasopharyngeal carcinoma), HT-29 (human colon carcinoma), MCF-7 (human breast carcinoma), A549 (human lung carcinoma), ASK (rat glioma), and non-cancerous Hek-293 cells (human embryonic kidney cells).

## Materials and methods

### General experimental procedures

The melting points were determined on a digital Electrothermal 9100. Optical rotations were measured with a JASCO DIP-370 digital polarimeter in a 50 mm microcell (1 mL). Ultraviolet and infra-red spectra were recorded using Shimadzu UV-2600 and Alpha Bruker spectrophotometers, respectively. Electron ionisation mass spectrometry (EI-MS) and high-resolution electrospray ionisation mass spectrometry (HR-ESI-MS) data were obtained on Thermo Finnigan Polaris Q and Micromass VQ-TOF2 spectrometers, respectively. ^1^H-NMR (400, 500 MHz), ^13^C-NMR (100, 125 MHz), and 2 D correlation spectra were recorded on Bruker Avance 500 and Bruker Ascend 400 NMR spectrometers in CDCl_3_, CD_3_OD, or C_5_D_5_N. Separation was achieved using silica gel P60 40–63 µm (SiliaFlash^®^; Silicycle), reversed phase silica gel C-18, 40–63 µm (Silicycle), and Sephadex™ LH-20 (GE Healthcare Life Sciences) as stationary phases for column chromatography and silica gel 60 F_254_ for preparative thin layer chromatography (PTLC, 20 × 20 cm, layer thickness 1 mm; Merck). Silica gel 60 PF_254_ (layer thickness 0.2 mm; Merck) TLC plates were used for analytical TLC. The TLC plates were detected by exposure to ultraviolet (UV) light at 254 and 366 nm and spraying with 30% H_2_SO_4_ in methanol, 1% CeSO_4_ in 10% aqueous H_2_SO_4_, or Dragendorff’s reagent.

### Plant materials

The leaves and stems of *M. sirikitiae* were collected from Mae Surin Waterfall National Park, Mae Hong Son Province, Thailand in September 2007. The plant was identified by Narong Nuntasaen and a voucher specimen (BKF144972) has been deposited at the Forest Herbarium, Royal Forestry Department, Bangkok, Thailand.

### Extraction, isolation, and characterisation

The air-dried powdered leaves (1.0 kg) and stems (2.0 kg) of *M. sirikitiae* were extracted by maceration with distilled MeOH (9 × 4 L), and the solvent was removed using a rotary evaporator, followed by freeze drying, to yield 102.0 g of crude MeOH leaf extract and 72.4 g of crude MeOH stem extract.

The crude MeOH leaf extract (90.0 g) was partitioned by dissolution in MeOH (100 mL) and deionized water (1.2 L). The aqueous suspension was sequentially partitioned into hexane, EtOAc, and *n*-BuOH to give hexane (20.7 g), EtOAc (21.1 g), *n*-BuOH (17.6 g), and aqueous (24.2 g) extracts, respectively. The hexane, and EtOAc extracts were combined according to their TLC characteristics and further separated using short column chromatography (CC) over silica gel using gradient elution with Me_2_CO–hexane and MeOH to give nine subfractions (A1–A9). The purification of subfraction A5 (8.66 g) by silica gel CC employing Me_2_CO–hexane and MeOH gradient elution led to eight subfractions (B1–B8). Subfraction B5 (1.36 g) was rechromatographed by silica gel CC by gradient elution with MeOH–CH_2_Cl_2_ to generate eight subfractions (C1–C8). Compound **1** (197.5 mg) was obtained as colourless crystals by recrystallization of subfraction C4. Subfraction A6 (7.71 g) was separated using silica gel CC and MeOH–CH_2_Cl_2_ gradient elution to give six subfractions (D1–D6). Subfraction D3 (1.98 g) was isolated by silica gel CC using gradient elution with Me_2_CO–hexane and MeOH to give six subfractions (E1–E6). Compound **1** (20.1 mg) was obtained as colourless crystals by the recrystallization of subfraction E2. The recrystallization of subfraction E4 led to the isolation of compound **2** (717.4 mg) as colourless crystals. Subfractions D4 (1.21 g) and E5 (126.0 mg) were combined and further separated by silica gel CC employing EtOAc–hexane and MeOH gradient elution to afford five subfractions (F1–F5). Subfraction F4 (279.3 mg) was rechromatographed on silica gel CC using MeOH–CH_2_Cl_2_ as a gradient mixture to afford four subfractions (G1–G4). The purification of subfraction G2 by recrystallization yielded compound **3** (24.0 mg) as colourless crystals. Subfraction A7 (7.10 g) was further separated by silica gel CC using gradient elution with CH_2_Cl_2_–hexane and MeOH–CH_2_Cl_2_ to yield eight subfractions (H1–H8). The recrystallization of subfraction H6 led to the purification of compound **4** (51.9 mg) as a white powder. Subfraction H2 (495.3 mg) was rechromatographed on silica gel CC using gradient systems of CH_2_Cl_2_–hexane and MeOH–CH_2_Cl_2_ to give four subfractions (I1–I4). Subfraction I2 (386.9 mg) was subjected to silica gel CC using MeOH–CH_2_Cl_2_ gradient elution to afford six subfractions (J1–J6). Compound **5** (13.9 mg) was obtained as yellow needles by the recrystallization of subfraction J3. Subfraction H3 (1.81 g) was rechromatographed using silica gel CC and gradient elution with Me_2_CO–hexane and MeOH to provide seven subfractions (K1–K7). Subfraction K3 (204.7 mg) was further separated by silica gel CC using gradient systems of CH_2_Cl_2_–hexane and MeOH–CH_2_Cl_2_ as eluents to yield six subfractions (L1–L6). Subfraction L2 (68.8 mg) was purified by Sephadex LH20 CC using MeOH as the eluent to yield three subfractions (M1–M3). The purification of subfraction M2 was conducted by recrystallization to give compound **6** (12.4 mg) as colourless crystals. Subfraction K4 (557.8 mg) was separated by silica gel CC using gradient elution with Me_2_CO–hexane as the mobile phase to give four subfractions (N1–N4). Subfraction N2 (414.1 mg) was further separated by silica gel CC using a gradient elution system of MeOH–CH_2_Cl_2_ to yield four subfractions (O1–O4). Subfractions O2 (156.6 mg) and O3 (181.5 mg) were isolated by Sephadex LH20 CC using MeOH as the mobile phase to afford three and four subfractions (P1–P3 and Q1–Q4), respectively. Subfractions P2 (136.1 mg) and Q2 (126.4 mg) were combined and further separated by silica gel CC using gradient elution with EtOAc–CH_2_Cl_2_ and MeOH–EtOAc to yield six subfractions (R1–R6). Compounds **7** (141.2 mg) and **8** (9.6 mg) were obtained as pale yellow semisolids from subfractions R2 and R5, respectively. Subfraction Q3 (30.4 mg) was purified by Sephadex LH20 CC and eluted with MeOH to give compound **9** (20.8 mg) as a pale yellow semisolid. Subfraction A8 (7.52 g) was isolated by silica gel CC employing MeOH–CH_2_Cl_2_ gradient elution to give eight subfractions (S1–S8). Subfraction S3 (260.8 mg) was further subjected to silica gel CC and was eluted with a gradient system of EtOAc–hexane and MeOH to yield seven subfractions (T1–T7). Subfraction T7 (78.3 mg) was purified over Sephadex LH20 CC using MeOH as the mobile phase to provide three subfractions (U1–U3). Compound **10** (8.7 mg) was obtained as yellow needles by recrystallization of subfraction U2, and the mother liquor was further separated by silica gel PTLC using 5% MeOH in CH_2_Cl_2_ as the mobile phase to give compound **10** (7.6 mg) as yellow needles and compound **11** (7.2 mg) as orange needles.

The crude MeOH extract of stems (60.0 g) was subjected to short CC over silica gel using a MeOH–CH_2_Cl_2_ gradient system to give six subfractions (A1–A6). Subfraction A1 (8.66 g) was further isolated by silica gel CC employing gradient systems of Me_2_CO-hexane and MeOH–Me_2_CO to yield five subfractions (B1–B5). After recrystallization, subfraction B1 yielded a mixture of compounds **12** and **13** (2.92 g) as colourless crystals. Subfraction A2 (9.56 g) was separated by silica gel CC using CH_2_Cl_2_–hexane and MeOH–CH_2_Cl_2_ gradient elution to afford nine subfractions (C1–C9). Subfraction C6 (4.57 g) was purified by silica gel CC using gradient elution with CH_2_Cl_2_–hexane and MeOH–CH_2_Cl_2_ to yield four subfractions (D1–D4). Subfraction D2 (3.51 g) was separated by silica gel CC employing CH_2_Cl_2_-hexane and MeOH–CH_2_Cl_2_ gradient elution to provide four subfractions (E1–E4). Subfraction E1 (1.53 g) was rechromatographed over silica gel CC with Me_2_CO–hexane and MeOH–Me_2_CO gradient elution to afford four subfractions (F1–F4). Compound **14** (13.7 mg) was obtained as a white powder by precipitation from subfraction F2. The mother liquor of subfraction F2 (1.19 g) was further purified by silica gel CC using gradient elution with Me_2_CO–hexane and MeOH–Me_2_CO to give five subfractions (G1–G5). The purification of subfraction G3 (378.4 mg) by recrystallization provided compound **1** (9.7 mg) as colourless crystals. Subfraction E2 (1.27 g) was purified by silica gel CC using gradient elution with MeOH–CH_2_Cl_2_ to yield five subfractions (H1–H5). The precipitate (72.6 mg) obtained from subfraction H3 was further purified by PTLC on silica gel and developed with 5% MeOH:CH_2_Cl_2_ to give compound **5** (12.4 mg) as yellow needles and compound **15** (5.1 mg) as a yellow powder. Subfraction D3 (629.1 mg) was separated by silica gel CC with MeOH–CH_2_Cl_2_ gradient elution to give four subfractions (I1–I4). Subfraction I3 (373.8 mg) was rechromatographed by Sephadex LH-20 CC using MeOH as the eluent to yield three subfractions (J1–J3). Subfraction J3 (55.3 mg) was purified by silica gel PTLC developed with 5% MeOH:CH_2_Cl_2_ to give compound **11** (12.2 mg) as orange needles. Subfraction A4 (12.37 g) was rechromatographed by silica gel CC using gradient elution with MeOH–CH_2_Cl_2_ to provide three subfractions (K1–K3). Subfraction K2 (7.53 g) was separated by acid-base extraction to generate an alkaloid subfraction (75.9 mg) which was further purified by silica gel PTLC developed with 15% MeOH:CH_2_Cl_2_ to yield compound **16** (10.9 mg) as yellow needles.

Mitrephoran (**6**): Colourless crystals were obtained from CH_2_Cl_2_/MeOH; m.p. 146–148 °C; ${\left[\alpha \right]}_{D}^{25}$ 14.00 (*c* 0.45, CHCl_3_); UV $\lambda_{\max}^{\mathrm{MeOH}}$ nm (log ε): 230 (4.37), 276 (4.13), 305 (3.93); Fourier transform infra-red (FTIR) spectroscopy (neat) ν_max_: 3396, 3259, 2954, 2872, 2841, 1672, 1597, 1587, 1515, 1463, 1439, 1418, 1357, 1347, 1287, 1259, 1158, 1122, 1088, 1060, 1033, 1020, 1005, and 968 cm^−1^; ^1^H-NMR (CDCl_3_, 400 MHz) and ^13^C-NMR (CDCl_3_, 100 MHz), see Table 1; HR-ESI-MS *m/z* 411.1420 [M + Na]^+^; EI-MS *m/z* 388 [M]^+^ (8), 218 (14), 196 (38), 180 (93), 165 (100), and 151 (60).

**Table 1.** ^1^H NMR (400 MHz), ^13^C NMR (100 MHz), and HMBC spectroscopic data of compound **6** in CDCl_3_.

Carbon	*δ*_C_ (ppm)	*δ*_H_ (ppm) (no. of proton, *mult*., *J* (Hz))	HMBC
2	83.91 (CH)	4.73 (1H, *d*, 9.1)	C-3, C-3a, C-1′, C-2′, C-6′
3	52.21 (CH)	2.96 (1H, *m*)	–
3a	61.44 (CH_2_)	3.73 (1H, *dd*, 10.9, 5.6) 3.83 (1H, *dd*, 10.9, 4.4)	C-2, C-3, C-4
4	49.67 (CH)	4.24 (1H, *m*)	C-2, C-3, C-3a, C-5
4a	198.04 (C=O)	–	–
5	70.85 (CH_2_)	4.24 (1H, *m*)	C-2, C-3
		4.37 (1H, *m*)	C-2, C-3, C-4
1′	132.31 (C)	–	–
2′	108.92 (CH)	7.08 (1H, *br s*)	C-2, C-1′, C-3′, C-4′, C-6′
3′	146.88 (C)	–	–
4′	145.63 (C)	–	–
5′	114.04 (CH)	6.93 (1H, *m*)*	C-1′, C-3′, C-4′
6′	120.17 (CH)	6.94 (1H, *m*)*	C-2, C-2′, C-4′
1″	129.77 (C)	–	–
2″	110.57 (CH)	7.63 (1H, *d*, 2.0)	C-3″, C-4″, C-6″
3″	149.26 (C)	–	–
4″	153.70 (C)	–	–
5″	110.12 (CH)	6.98 (1H, *d*, 8.4)	C-1″, C-3″, C-4″
6″	123.23 (CH)	7.67 (1H, *dd*, 8.4, 2.0)	C-2″, C-4″, C-5″
3′-OMe	56.02 (CH_3_)	3.99 (3H, *s*)	C-3′
3″-OMe	56.06 (CH_3_)	4.01 (3H, *s*)	C-3″
4″-OMe	56.18 (CH_3_)	4.02 (3H, *s*)	C-4″

*H-5′ and H-6′ were observed as broad singlets in the ^1^H-NMR spectrum due to the virtual coupling effects of the *ortho* protons which are close in chemical shifts.

### X-ray crystal structure analysis

X-ray crystallographic data for C_21_H_24_O_7_, *M_W_* = 388.42, 0.35 × 0.30 × 0.30 mm, orthorhombic space group *P*2_1_2_1_2_1_ (No. 19). *a* = 9.5519(9) Å, *b* = 9.7662(10) Å, *c* = 21.087(2), *α* = *β* = *γ* 90.00°, *V* = 1967.1(3) Å^3^, *Dx* = 1.311 g cm^−3^, *Z* = 4, *F*_(000)_ = 824. A total of 3733 reflections, of which 8526 were unique (3581 observed, *I* > 2σ(*I*)) *T* = 273(2) K, μ (Cu *K*α) = 0.82 mm^−1^, reflections collected/unique: 15905/9859, number of observations [*I* > 2σ(*I*)]: 3581, *R*_1_ = 0.0276, *wR*_2_ = 0.0739 (all data), Flack parameter = 0.01(11). X-ray crystallographic data were measured using a graphite monochromatic Cu *K*_α_ radiation source (λ = 1.54178 Å) on a Bruker D8 VENTURE diffractometer. The structure was solved by using Olex2 (Dolomanov et al. 2009) and refined with full-matrix least-squares calculations on *F^2^* using SHELXL-2015 (Sheldrick 2015). The crystallographic data were deposited at the Cambridge Crystallographic Data Centre under reference number CCDC1962628.

### *In vitro* cytotoxic activity assay

The methanol extracts and the isolated compounds were evaluated for their cytotoxic activities against several cell lines, including murine lymphocytic leukaemia (P-388), human oral epidermoid carcinoma (KB), human colon carcinoma (Col-2 and HT-29), human breast cancer (MCF-7), human lung carcinoma (Lu-1 and A549), rat glioma (ASK), and noncancerous human embryonic kidney cell (HEK-293) by sulforhodamine B (SRB) assay in 96-well microtiter plates (Skehan et al. 1990). This method measures the cellular protein content of cultures in 96-well microtiter plates. The cell lines were seeded into 96-well microtiter plates and treated with the test compounds at concentrations of 0.16–20 µg/mL for 72 h, except for the P-388 cells, which were treated for 48 h. Then the cell cultures were fixed with 20% trichloroacetic acid (Merck) and stained with 0.4% SRB (Sigma-Aldrich) dissolved in 1% acetic acid (Merck) for 1 h. The cellular-protein-bound dye was extracted with 10 mM unbuffered Trisbase solution (pH 10.5) (Sigma-Aldrich) for the determination of optical density at 510 nm with a microtiter plate reader (Thermo Scientific™ Multiskan™ GO Microplate Spectrophotometer). The cytotoxic potency was expressed as median inhibition concentrations (IC_50_), the concentration that inhibits 50% of cell viability (µg/mL and µM). The IC_50_ values were determined from the nonlinear regression curve fit in GraphPad Prism software (version 5). Ellipticine (Sigma-Aldrich), a cytotoxic plant alkaloid causing topoisomerase II inhibition and DNA intercalation, was used as a positive control.

## Results

### Isolation and characterisation

**Figure 1.**
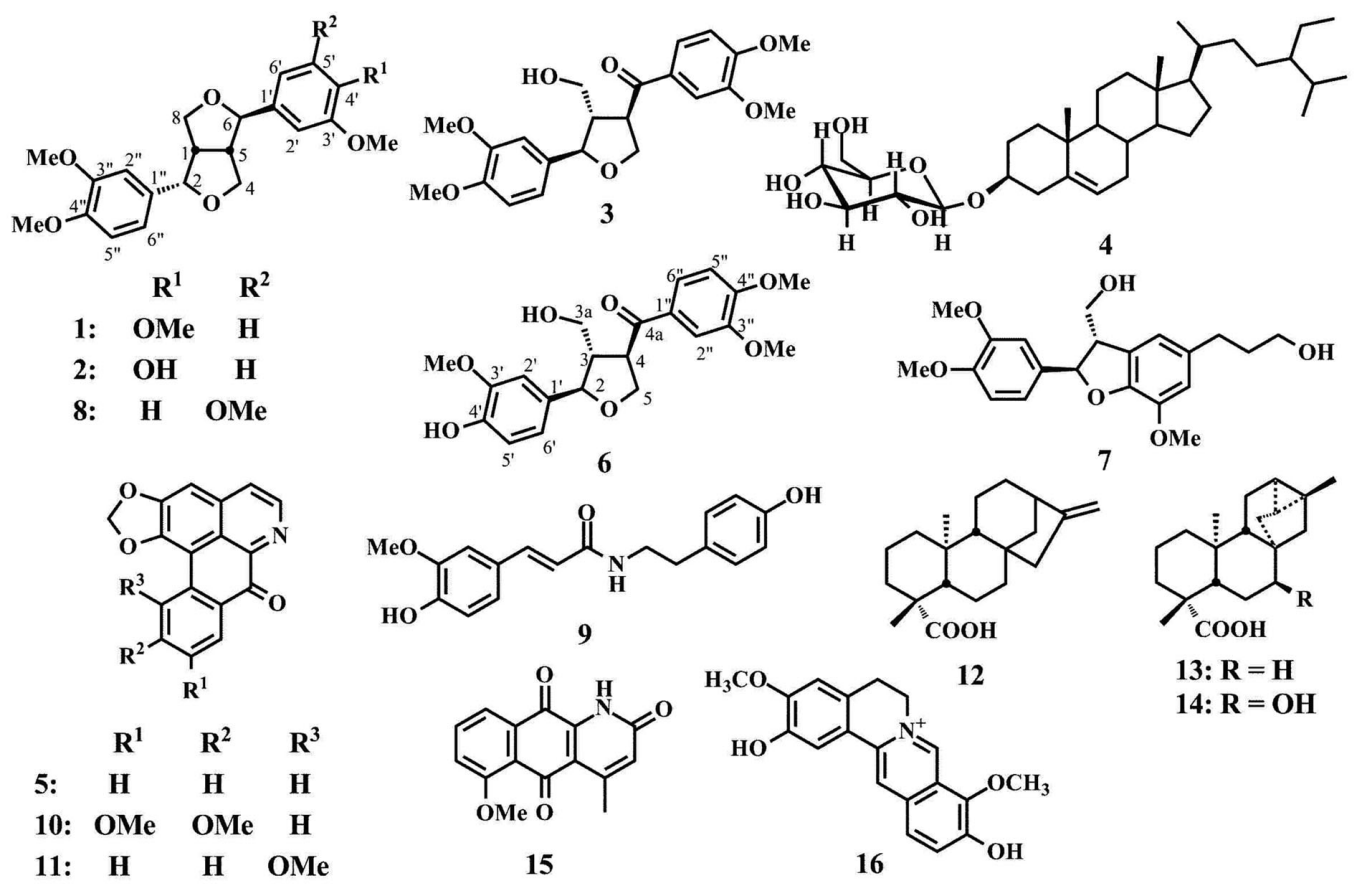
The isolated compounds from the leaves and stems of *Mitrephora sirikitiae*.

The isolation of the leaf extract using chromatographic techniques and recrystallization resulted in the isolation of a new lignan, mitrephoran (**6**), together with five known lignans **1**–**3**, **7**, and **8**, one known steroidal glycoside **4**, and four known alkaloids **5**, **9**, **10**, and **11**. Moreover, the separation of the stem extract also yielded compounds **1**, **4**, **5**, and **11** (as for the leaf extract), together with three known diterpenoids **12**–**14**, and two known alkaloids **15** and **16**. Compounds **1**–**5** and **7**–**16** were identified as (–)-epieudesmin (**1**) (Kaku and Ri 1937; Ahmed et al. 2002), (–)-phylligenin (**2**) (Rahman et al. 1990), magnone A (**3**) (Jung et al. 1998), stigma-5-en-3-*O*-*β*-glucopyranoside (**4**) (Faizi et al. 2001), liriodenine (**5**) (Zhang et al. 2002; Chiu et al. 2012), 3′,4-*O*-dimethylcedrusin (**7**) (Pieters et al. 1993; Rayanil et al. 2016), 2-(3,4-dimethoxyphenyl)-6-(3,5-dimethoxyphenyl)-3,7-dioxabicyclo[3.3.0]octane (**8**) (Wang et al. 2012), *N*-*trans*-feruloyltyramine (**9**) (Kim et al. 2005), dicentrinone (**10**) (Zhou et al. 2000), oxoputerine (**11**) (Lu et al. 2011), kaurenoic acid (**12**) (Batista et al. 2007), *ent*-trachyloban-19-oic acid (**13**) (Leong and Harrison 1997; Ngamrojnavanich et al. 2003), ciliaric acid (**14**) (Ngouela et al. 1998), 6-methoxymarcanine A (**15**) (Tsai and Lee 2010), and stepharanine (**16**) (Ingkaninan et al. 2006) (Figure 1) based on their IR, UV, NMR, and MS spectroscopic data and by comparison with previously reported spectroscopic and physical data.

**Figure 2.**
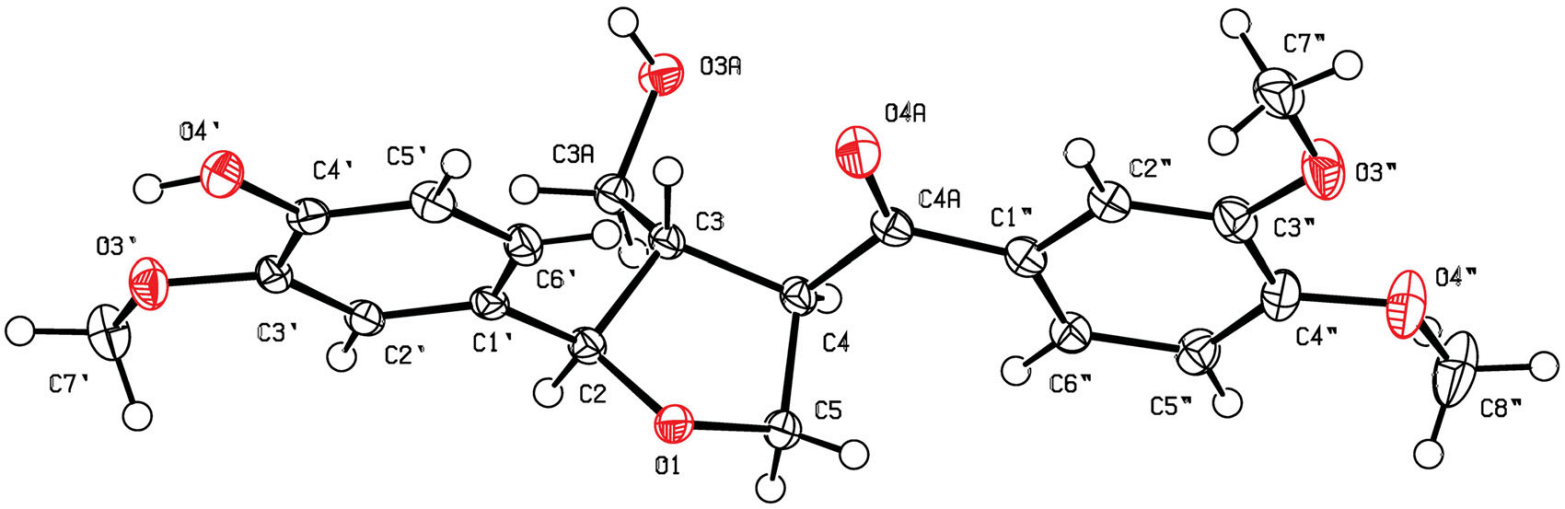
The ORTEP view by X-ray diffraction analysis of compound **6.**

Mitrephoran (**6**) was isolated as colourless crystals. The HR-ESI-MS data revealed the parent ion at *m/z* 411.1420 [M + Na]^+^, suggesting a molecular formula of C_21_H_24_O_7_. Moreover, in the EI-MS spectrum of **6**, the molecular ion and base ion peaks were observed at *m/z* 388 [M]^+^ (8) and 165 (100) which indicate benzylic and tetrahydrofuran ring fragments. The IR spectrum of **6** showed the characteristic absorption bands of a hydroxyl group and a carbonyl group conjugated with an aromatic ring at 3396 and 1672 cm^−1^. In addition, its UV spectrum contained λ maxima at 230, 276, and 305 nm, corresponding to the furanoid lignan skeleton. The ^13^C and distortionless enhancement by polarisation transfer (DEPT) NMR spectra of **6** indicated the presence of 21 carbons atoms, including one carbonyl carbon, six aromatic methine carbons, six aromatic quaternary carbons, three methane carbons, two methylene carbons, and three methoxy carbons, as listed in Table 1. The ^1^H-NMR spectrum of **6** showed three singlets at *δ* 3.99, 4.01, and 4.02 ppm indicating the presence of three methoxy substituents on aromatic rings at C-3′, C-3″, and C-4″, respectively. The six signals in the low-field region of the ^1^H NMR spectrum were assigned to six aromatic protons of two 1,3,4-trisubstituted benzene rings. The first set appearing as an ABX coupling system consisting of *δ* 7.67 (1H, *dd*, 8.4, 2.0 Hz, H-6″), 7.63 (1H, *d*, 2.0 Hz, H-2″), and 6.98 (1H, *d*, 8.4 Hz, H-5″), and these were assigned to the protons of a 3″,4″-dimethoxyphenyl ring connected with a carbonyl carbon (C-4a). The broad singlets at *δ* 7.08 (1H, *s*, H-2′), 6.94 (1H, *s*, H-6′), and 6.93 (1H, *s*, H-5′) were identified to three aromatic protons on a 3′-methoxy-4′-hydroxyphenyl ring. The proton signals at *δ* 3.83 (1H, *dd*, 10.9, 4.4 Hz, H-3a), 3.73 (1H, *dd*, 10.9, 5.6 Hz, H-3a), and 4.37 and 4.24 (each 1H, *m*, H-5) were assigned to non-equivalent methylene protons on carbon atoms bearing oxygen atoms of a hydroxymethylene substituent and a furan ring, respectively. The ^1^H-NMR spectrum of **6** also displayed signals at *δ* 4.73 (1H, *d*, 9.1 Hz, H-2), 2.96 (1H, *m*, H-3), and 4.24 (1H, *m*, H-4) assignable to the three methine protons at C-2, C-3, and C-4, respectively. Full assignment of the 1 D NMR spectra and the connectivities of compound **6** were established based on ^1^H-^1^H correlated spectroscopy (COSY), heteronuclear single quantum coherence spectroscopy (HSQC), and heteronuclear multiple bond correlation (HMBC) spectroscopic data analyses. Moreover, the absolute configuration and positions of three methoxy and one hydroxy functional groups were confirmed by single-crystal X-ray diffraction analysis. The NMR spectra of compound **6** are similar to that of forsylthialan B (Piao et al. 2008), but the HMBC spectroscopic and X-ray crystallographic data indicated that there is a hydroxyl group at C-4′ of the phenyl ring, and stereocenters carbons C-2, C-3, and C-4 were assigned as *R*, *S*, and *R* configurations, respectively (Figure 2).

### *In vitro* cytotoxic activity

The cytotoxicity testing of the methanol extracts from the leaves, and stems of *M. sirikitiae* revealed that the leaf extract exhibited moderate to potent cytotoxicity against P-388, KB, Col-2, MCF-7, and Lu-1 cell lines, having IC_50_ values of 0.7, 5.1, 12.0, 2.6, and 3.9 µg/mL, respectively. In contrast, the stem extract showed lower cytotoxicity against P-388, MCF-7, and Lu-1, having IC_50_ values of 7, 13, and 17 µg/mL, respectively. Therefore, the extracts were further investigated with respect to the component cytotoxins. The isolated compounds (**1**–**16**) were subjected to cytotoxic activity assays against cancer cell lines: P-388, KB, HT-29, MCF-7, A549, and ASK and non-cancerous Hek-293 cells, and the results are presented in Table 2. Among those compounds, the alkaloids liriodenine (**5**) and oxoputerine (**11**) exhibited potent cytotoxic activity against all tested cell lines, having IC_50_ values in the range of 6.59–11.02 µM. 6-Methoxymarcanine A (**15**) inhibited the growth of four tested cell lines, P-338, HT-29, MCF-7, and A549, having IC_50_ values ranging from 8.33 to 12.30 µM but showed low cytotoxicity for KB and ASK cancer cell lines and the non-cancerous Hek-293 cell line. The lignan magnone A (**3**) showed specific cytotoxicity against P-388 and MCF-7, having IC_50_ values of 8.96 and 4.40 µM, respectively. 3′,4-*O*-Dimethylcedrusin (**7**) exhibited moderate to strong cytotoxic effects on KB, MCF-7, and A549 cells, having IC_50_ values of 2.03, 3.77, and 10.32 µM, respectively, but it was also toxic to the Hek-293 non-cancerous cell line. Therefore, magnone A (**3**) and 6-methoxymarcanine A (**15**) are potentially useful anticancer agents because of their specific inhibitory activities in some tested cancer cell lines and non-toxic effects on non-cancerous cells. In contrast, the other compounds were inactive against all tested cell lines.

**Table 2.** Cytotoxic activity of the isolated compounds from the leaves and stems of *Mitrephora sirikitiae*.

Compounds	Cell Lines / IC_50_ (*μ*M)						
	P-388	KB	HT-29	MCF-7	A549	ASK	Hek-293
**1**	–	–	–	–	–	–	–
**2**	32.50 ± 2.04	–	–	36.08 ± 1.02	–	–	–
**3**	8.96 ± 0.70	20.55 ± 3.88	27.61 ± 3.53	4.40 ± 0.10	37.94 ± 5.42	–	23.16 ± 1.17
**4**	–	–	–	–	–	–	–
**5**	9.60 ± 0.58	11.02 ± 0.11	10.62 ± 0.36	9.20 ± 0.25	9.45 ± 0.18	10.65 ± 0.95	8.07 ± 0.11
**6**	–	–	–	28.63 ± 0.15	–	–	–
**7**	25.88 ± 2.99	2.03 ± 0.11	19.73 ± 1.52	3.77 ± 0.05	10.32 ± 2.49	–	8.48 ± 4.04
**8**	–	15.88 ± 0.36	–	21.94 ± 2.96	–	–	16.51 ± 0.94
**9**	–	–	–	–	–	–	–
**10**	–	–	–	36.93 ± 1.13	–	–	–
**11**	6.72 ± 0.36	7.67 ± 0.30	7.28 ± 0.23	7.05 ± 0.20	7.21 ± 0.79	6.59 ± 0.43	7.31 ± 0.07
**12 + 13**	–	–	–	–	–	–	–
**14**	–	–	–	–	–	–	–
**15**	9.55 ± 2.83	38.07 ± 0.52	10.15 ± 0.07	8.33 ± 0.63	12.30 ± 0.37	38.69 ± 1.12	–
**16**	–	–	–	–	–	–	–
Ellipticine	2.03 ± 0.08	2.36 ± 0.04	2.40 ± 0.04	2.15 ± 0.08	2.64 ± 0.24	2.56 ± 0.04	2.68 ± 0.08

## Discussion

The isolated lignans **1**–**3** and **7**, alkaloids **5** and **9**–**11**, and diterpenoids **12**–**13** have also been also isolated from the other species in the genus *Mitrephora* (Zgoda-Pols et al. 2002; Yu et al. 2005; Deepralard et al. 2007; Ge et al. 2008; Li et al. 2009; Moharam et al. 2010, 2014; Rayanil et al. 2016). In contrast, lignan **8**, diterpenoid **14**, and alkaloids **15** and **16** were isolated from this genus for the first time, although they have been found already in plants in the family Annonaceae (Ngouela et al. 1998; Nishiyama et al. 2004; Tsai and Lee 2010; Wang et al. 2012).

Various natural alkaloids are highly cytotoxic against many cancer cell lines via various different mechanisms of action, and many of these compounds have been developed into anticancer drugs such as vinblastine, vincristine, camptothecin, taxol, and ellipticine (Isah 2016; Iqbal et al. 2017). Liriodenine (**5**) and oxoputerine (**11**), aporphine alkaloids, have been reported in many plants of the family Annonaceae. These compounds exhibit cytotoxicity against cancer cell lines A549 (human lung carcinoma), BGC-823 (human gastric carcinoma), BEL-7402 (human liver carcinoma), HTC-8 (human colon carcinoma), and A2780 (human ovarian carcinoma) (Lu et al. 2011). Moreover, the anticancer property of liriodenine (**5**) is related to its anti-proliferative, apoptosis-inducing (Nakano et al. 2013), and topoisomerase II inhibitory effects (Woo et al. 1999). Liriodenine (**5**) also arrests the cell cycle by increasing in intracellular nitric oxide (NO) production, the overexpression of apoptosis-related proteins, p53 (Chen et al. 2012) and Bax, and the suppression of Bcl-2 (Nordin et al. 2015). However, there are no reports of the cytotoxicity of 6-methoxymarcanine A (**15**), a rare natural 1-azaanthraquinone alkaloid. However, it was revealed that marcanine A, a derivative of compound **15**, exhibited cytotoxicity against various cancer cell lines A-549, HT-29, MCF-7, RPMI (melanoma), and U251 (human brain carcinoma) (Soonthornchareonnon et al. 1999).

Some types of lignans have been reported to have cytotoxic and anticancer activities. For example, sesamin is a furofuran-type lignan and is used as a dietary supplement because of its pharmacological activity, including protective effects against oxidative stress and cancer. The anticancer properties of sesamin are associated with its anti-proliferative, pro-apoptotic, anti-inflammatory, anti-metastatic, anti-angiogenic, and pro-autophagocytic effects (Majdalawieh et al. 2017). In addition, aryltetralin lactone lignans such as podophyllotoxin and its derivatives have been shown to possess anticancer and antitumor activities because of their tubulin polymerisation and DNA topoisomerase II inhibitory activities (Ardalani et al. 2017). Moreover, 2,3-naphtalide lignans (Fukamiya and Lee 1986; Jin et al. 2014), butane-type lignans (Lambert et al. 2005; Wukirsari et al. 2014), and dihydrobenzofuran neolignans (Huang et al. 2013; Fukui et al. 2018) have been found to be cytotoxic against many cancer cell lines. The natural dihydrobenzofuran neolignan 3′,4-*O*-dimethylcedrusin (**7**) has been shown to possess anticancer activity by inhibition of cell proliferation (Pieters et al. 1993). It has also been revealed that synthetic benzofuran lignan derivatives can mediate cell death by the induction of G2/M cell cycle arrest via a p53-dependent pathway (Manna et al. 2010). Furthermore, in our study, the tetrahydrofuran lignan **3** showed cytotoxicity against some tested cancer cell lines. A comparison of the activity of compounds **3** and **6** indicates that the methoxy group at C-4′ of the aromatic ring is important to the activity of these compounds.

## Conclusions

Chemical studies of *M. sirikitiae* leaves and stems led to the isolation of one new lignan, mitrephoran (**6**) and 15 known compounds, including five lignans (**1**–**3** and **7**–**8**), six alkaloids (**5**, **9**–**11**, **15**–**16**), one steroidal glycoside (**4**), and three diterpenoids (**12**–**14**). Among these compounds, the alkaloids liriodenine (**5**) and oxoputerine (**11**) were found to have potent cytotoxic activity against all tested cell lines. The lignans magnone A (**3**) and 3′,4-*O*-dimethylcedrusin (**7**), together with the alkaloid 6-methoxymarcanine A (**15**), showed specific cytotoxicity against some tested cell lines. Moreover, compounds **3** and **15** showed low toxicity towards non-cancerous cells. In summary, the lignans and alkaloids from *M. sirikitiae* leaves and stems, especially magnone A (**3**) and 6-methoxymarcanine A (**15**), have interesting anticancer activity and should be further investigated to determine their mechanisms of action.
